# Estimating the Potential of Insects from Warmer Regions to Overwinter in Colder Regions under a Warming Winter Scenario Using Simulation Experiments: A Case Study in *Sesamia nonagrioides*

**DOI:** 10.3390/insects14120957

**Published:** 2023-12-17

**Authors:** Jan Rozsypal

**Affiliations:** Biology Centre CAS, Institute of Entomology, Branišovská 31, 370 05 České Budějovice, Czech Republic; rozsypal@entu.cas.cz; Tel.: +420-387-775-231

**Keywords:** winter climate change, range shifts, simulation experiment, insect pests, *Sesamia nonagrioides*

## Abstract

**Simple Summary:**

Ongoing climate change is causing temperatures to rise in both summer and winter, allowing insect pests to invade new areas and potentially causing economic and human health problems. Low winter temperatures are thought to be one of the main barriers to the colonization of higher latitudes. Climate models predict that winter temperatures will increase more than summer temperatures in temperate regions, which may allow insects from warmer regions to colonize the colder, higher latitudes in the future. Understanding how climate change will affect insect distributions is critical for many areas of human activity. This paper presents a method to assess the potential of insects to colonize colder regions under a warming winter scenario. The method is based on exposing insects to laboratory simulations of a warming winter climate. The applicability of the method is tested using the example of a Mediterranean pest, *Sesamia nonagrioides*, whose ability to colonize Central Europe is assessed. The results indicate that *S. nonagrioides* could survive Central European winters even under the current state of warming or under a warmer climate predicted for the near future. The presented method may be particularly useful in pest management to estimate overwinter survival and distribution of pests due to climate change.

**Abstract:**

Ongoing climate change and anthropogenic pressure are having a profound influence on insects, causing species diversity to decline and populations to shrink. Insect pests invade new areas and cause economic and human health problems. Low temperatures in winter are thought to be one of the main barriers to the successful colonization of higher latitudes. Climate models predict that winter temperatures will increase more than summer temperatures in temperate and polar regions, potentially allowing species from warmer climates to colonize higher latitudes. Understanding how climate change will affect the distribution of insects is critical to many areas of human activity. One possible but seldom used way to predict likely range shifts of insects due to climate change is through simulation experiments. Here, I present and test a method to assess the potential of insect species from warmer regions to survive winters in colder regions under a warming winter scenario. The method is based on laboratory simulations of warming winters. The applicability of the method is demonstrated using the example of a Mediterranean pest, *Sesamia nonagrioides*, whose ability to survive Central European winters under a warming winter scenario is assessed. The method presented here is relatively simple, with potentially high accuracy of estimates.

## 1. Introduction

Insects represent one of the largest groups of animals on Earth and have colonized a wide range of environments [1]. Ongoing climate change and anthropogenic pressure, particularly in recent decades, appear to have had unprecedented effects on insect populations. Most notable are changes in the geographic distribution of insect species, an overall decline in diversity, and shrinking insect populations [2,3,4]. Insect pests are shifting their range and invading new areas, which can cause economic problems and also have negative effects on human health [5,6,7].

Efforts have been underway for some time to predict climate change-induced range shifts of economically and otherwise important insect species, such as pests of agricultural crops. It is widely accepted that the distribution of insect species is largely determined by their tolerance to abiotic stress [8]. Low winter temperatures are thought to be one of the main barriers to the successful colonization of higher-latitude regions [9,10]. Climate models predict that winter temperatures will rise more than summer temperatures in temperate and polar regions [11], which may allow insect species from warmer regions to colonize the colder, higher latitudes in the future [3].

There are several ways to study and possibly predict the effects of climate change on insect distributions. The most obvious, but also the most impractical, is to simply wait and see what happens. Currently, the most common approach to predicting changes in species distributions is to develop predictive models [12]. However, the most complex predictive models require the input of various parameters that are not always easy to obtain. Another way to predict changes in species distributions is through simulation experiments. At first glance, simulation experiments may seem even more complex than predictive models, which may be the main reason why simulations are rarely (if at all) used for this purpose. However, the use of a simulation approach could be advantageous because organisms are directly involved in the experiments. This could potentially allow for a more accurate assessment of the parameters being studied and their effects, as well as highlight the influence of factors that would normally not be considered.

Here, I present and test a method to predict the potential of a species from a warmer region to survive winters in a colder region under a warming winter scenario. My method is based on the simulation experiment approach, which directly exposes the studied organisms to simulated environmental conditions. I test the feasibility of the simulation approach using the Mediterranean corn borer (*Sesamia nonagrioides*) (Lefèbvre) (Lepidoptera: Noctuidae) as a model.

*Sesamia nonagrioides* is a major pest of maize in the Mediterranean region. It originates from tropical regions but has expanded its range to subtropical and warm temperate climates of the Palearctic region over the last ca. 100,000 years. Within the Palearctic region, its distribution extends from the Mediterranean Basin to the Caspian Sea and does not exceed the 45° parallel [13,14]. It overwinters in a larval stage in diapause, which it enters in response to a photoperiodic signal [15]. It has a limited capacity for cold acclimation and can survive at temperatures above its supercooling point [16,17].

This “pilot” study simulates changes in a single (but probably the most important) parameter—temperature. The simulations are used to assess the ability of a Spanish population of *S. nonagrioides* to survive Central European winters as they become warmer due to climate change. The insects are thus exposed to simulated warm winter scenarios in the laboratory. The simulations are based on long-term average weekly temperatures occurring in the target geographic area of interest (Czechia in this study). The long-term averages are increased by a certain value in each scenario (simulating warming), and the effect on survival is assessed. Simply put, the method is designed to answer questions such as, “Does species X have a chance of surviving winters in region Y if they warm by Z °C?” Consequently, this method cannot be employed as a tool for general predictions of the effects of climate change on insects, but it could be successfully applied to specific species of interest, such as pests.

In addition to the laboratory simulations, the insects in this study were exposed to three consecutive real winters in Central Europe (Czechia) under semi-natural conditions in the field. The field experiment shows how different real winters affect survival and supports the results of the laboratory simulations.

## 2. Materials and Methods

### 2.1. Insects

Laboratory culture of *S. nonagrioides* was initiated from larvae collected in the field in autumn 2019 in Lleida, Spain. Adults (i.e., moths) were kept in cages with young maize plants for oviposition and had access to water. Maize plants were grown from seeds purchased at a garden store. Larval stages were kept in incubators at 25 °C and 16L:8D photoperiod. The larvae were reared in plastic containers (250 mL) on a semi-artificial diet based on Eizaguirre & Albajes [15]. One kilogram of the diet consisted of the following components: 15 g of agar, 102 g of corn polenta, 27.5 g of wheat germ, 27.5 g of yeast, 2 g of potassium sorbate, 1 g of methyl 4-hydroxybenzoate, 5 g of ascorbic acid, 1/2 tablet of vitamin B complex (B-komplex forte, Generica, Slovakia), and 820 mL of water. Agar, corn polenta, wheat germ, and yeast were mixed with water and brought to a boil. After cooling to ca. 60 °C, the remaining components were added. The diet was changed weekly. The population density was approximately 100 individuals per 100 g of the diet in the first instar larvae and was gradually reduced to approximately 20 individuals per 100 g of the diet in the sixth (i.e., last) instar larvae.

Larvae intended for experiments were reared on the same diet and temperature as the larvae in the culture but under diapause-inducing photoperiod of 10L:14D [18] and were cold-acclimated after reaching the last instar. Cold acclimation was performed in four steps by exposing the larvae to gradually decreasing temperatures. The larvae were first exposed to 20 °C, 15 °C, and 10 °C, each temperature for 7 days, while food was still available. In the last step, the larvae were exposed to 8 °C for 3 days without access to food. After cold acclimation, the larvae were transferred to containers made of Plexiglas cylinders (height = 110 mm, inner diameter = 62 mm, wall thickness = 4 mm) closed with perforated lids on both sides and filled with strips of corrugated cardboard. The containers with the larvae were then exposed either to the simulated winter scenarios in incubators or in the field. The containers were designed to minimize the risk of accidental release of the insects into the wild.

### 2.2. Treatments

**Figure 1:**
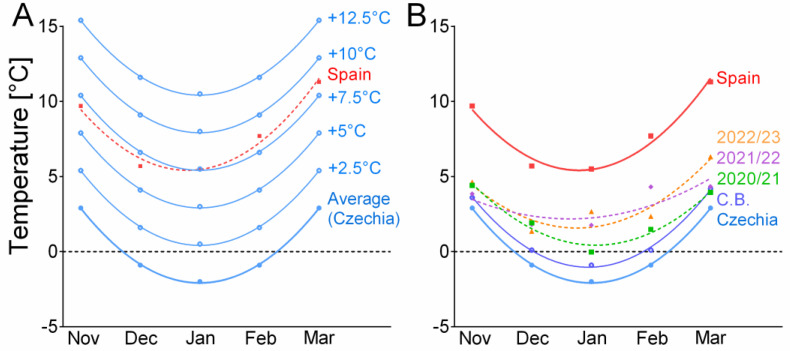
Laboratory and field air temperatures. The figure shows the temperatures in the laboratory scenarios and the monthly mean air temperatures in the field. (**A**) shows laboratory warming scenarios (i.e., simulations) that were derived from the monthly means in Czechia in the period from 1981 to 2010 (Czechia = lab “Average”) by increasing the “Average” temperatures in 2.5 °C steps up to the “Average + 12.5 °C”. The dashed red line shows long-term mean temperatures (1981–2010) in Spain (Lleida; place of origin of the lab culture of *S. nonagrioides*) for comparison. (**B**) shows monthly mean air temperatures calculated from our own temperature records (recorded in the shade 2 m above the ground during winter seasons 2020/21, 2021/22, 2022/23), compared to the long-term means (1981–2010) in Czechia and Spain (Lleida). The figure also includes mean temperatures for the same period for České Budějovice (C.B.). The long-term means were obtained from the Czech Hydrometeorological Institute (Czechia) and the State Meteorological Agency (Spain). Second-order polynomial curves were fitted to the monthly means (goodness of fit, R2; Czechia/Average/Average + X°C = 0.999; C.B. = 0.997; Spain = 0.977; 2020/21 = 0.968; 2021/22 = 0.639; 2022/23 = 0.867.

The laboratory warming scenarios (Figure 1a, Supplementary Table S3) were designed to test how warming winter climate will affect survival. The baseline “Average” scenario simulated a long-term average winter in Czechia, based on weekly means from 1981 to 2010. The laboratory scenarios simulated a period from the beginning of November to the end of March. The warming scenarios were modifications of the “Average” scenario that was warmed in 2.5 °C steps up to the “Average + 12.5 °C”. The range of simulated temperatures was chosen arbitrarily to test the method. However, the “A + 2.5 °C” scenario corresponds relatively well with the upper estimates of climate models for the 2040s, and the “A + 5 °C” scenario corresponds well with the upper estimates for the end of the 21st century in Czechia [19]. Scenario “A + 7.5 °C” is close to the long-term (1981-2010) mean temperatures in Lleida, Spain (the origin of the laboratory population of *S. nonagrioides* used in this study). The simulations included a natural-like progression of the light/dark cycle at latitude of 50° N. The simulations were performed in Sanyo MIR 154 incubators (Sanyo Electric, Osaka, Japan). Temperature and photoperiod in all laboratory scenarios were changed in weekly steps (see Supplementary Table S3 for details). Relative humidity in incubators was maintained above ca. 60% with containers of distilled water holding a folded filter paper (to promote evaporation). Three to six groups (i.e., replicates) of larvae were exposed to each scenario. There were 40 or 48 larvae in each group (see Supplementary Table S1 for exact numbers).

**Figure 2:**
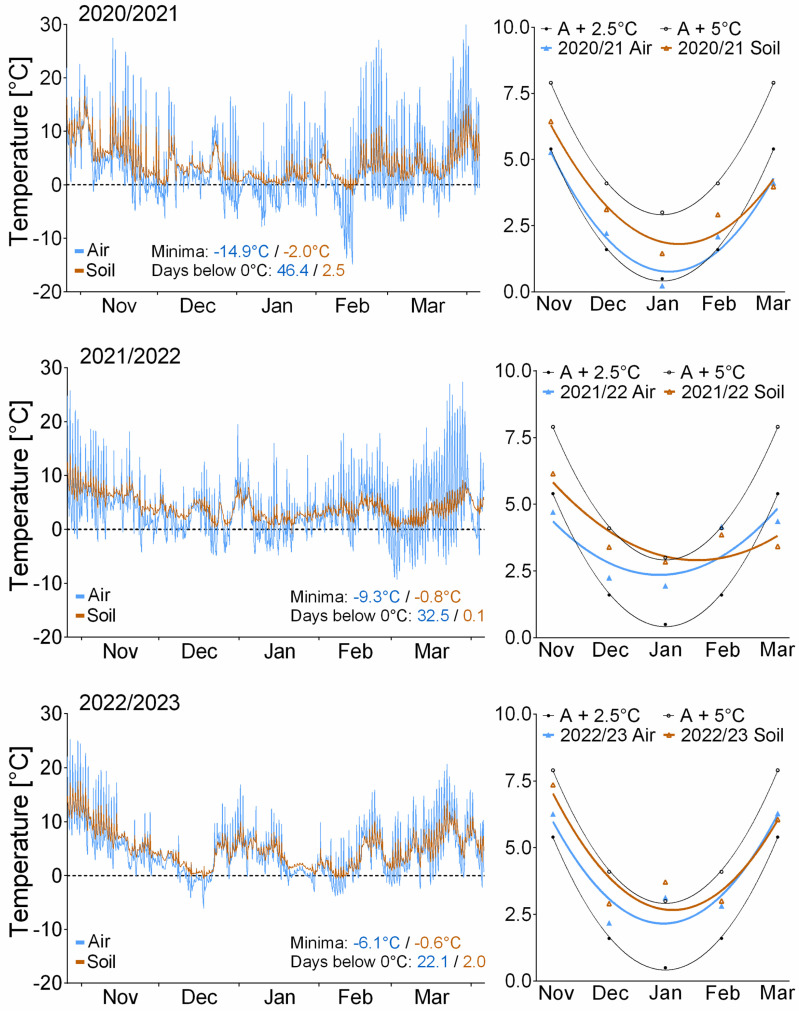
Microclimatic temperatures. The left column of the figure shows microclimatic temperatures recorded during the field experiments in winter seasons 2020/2021, 2021/2022, and 2022/2023. The probes (thermocouples) were placed in the same conditions experienced by the larvae. The figures also show the absolute minima and the sum of time (in days) below 0 °C. The right column of the figure shows monthly mean temperatures in microclimates compared to laboratory scenarios “Average + 2.5 °C” and “Average + 5 °C”. The monthly means were calculated from our own temperature records (Table S4; left part of Figure 2). Second-order polynomial curves were fitted to the monthly means (goodness of fit, R2; Average + X °C = 0.999; 2020/21 Air = 0.956; 2020/21 Soil = 0.942; 2021/22 Air = 0.687; 2021/22 Soil = 0.788; 2022/23 Air = 0.873; 2022/23 Soil = 0.856.

The field treatments exposed the insects to semi-natural conditions in the field in České Budějovice, Czechia. The insects were exposed for part of the year, corresponding to the simulations (i.e., from November to March). The larvae in containers were exposed either above ground (on the soil surface, exposed to near-air temperatures) or below ground (10 cm deep, exposed to soil temperatures), taking into consideration that part of the larvae overwinters in maize stalks above and part below the ground [16]. The temperature experienced by the larvae was measured by thermocouples connected to data logger Testo 176 T4 (Testo, Titisee-Neustadt, Germany) (for temperature records, see Figure 2 and Supplementary Table S4). The field experiment was repeated three times during winter seasons of 2020/2021, 2021/2022, and 2022/2023. Each winter season, three groups (i.e., replicates) of larvae were exposed to both treatments (“Air” or “Soil”). There were 40, 45, or 50 larvae in each group (see Supplementary Table S1 for exact numbers).

### 2.3. Analysis of Survival

At the end of the experiments, the insects were transferred to permissive conditions (25 °C and 16L:8D photoperiod), and survival was analyzed. First, the ability to move was assessed 24 h after transfer to the permissive conditions. The larvae were judged alive when capable of coordinated movement. Second, the ability to pupate was assessed. Larvae were kept in permissive conditions until they either pupated or died.

### 2.4. Statistical Analysis

A non-parametric Kruskal–Wallis test was used to analyze the effect of treatment on the measured parameter. Dunn’s post hoc test was used to find the differences among treatments. The Mann–Whitney test was used to analyze the differences between two groups. Statistical calculations were performed using Prism v.6 (Graphpad Software, San Diego, CA, USA).

## 3. Results

### 3.1. Survival in the Laboratory Scenarios and in the Field

**Figure 3:**
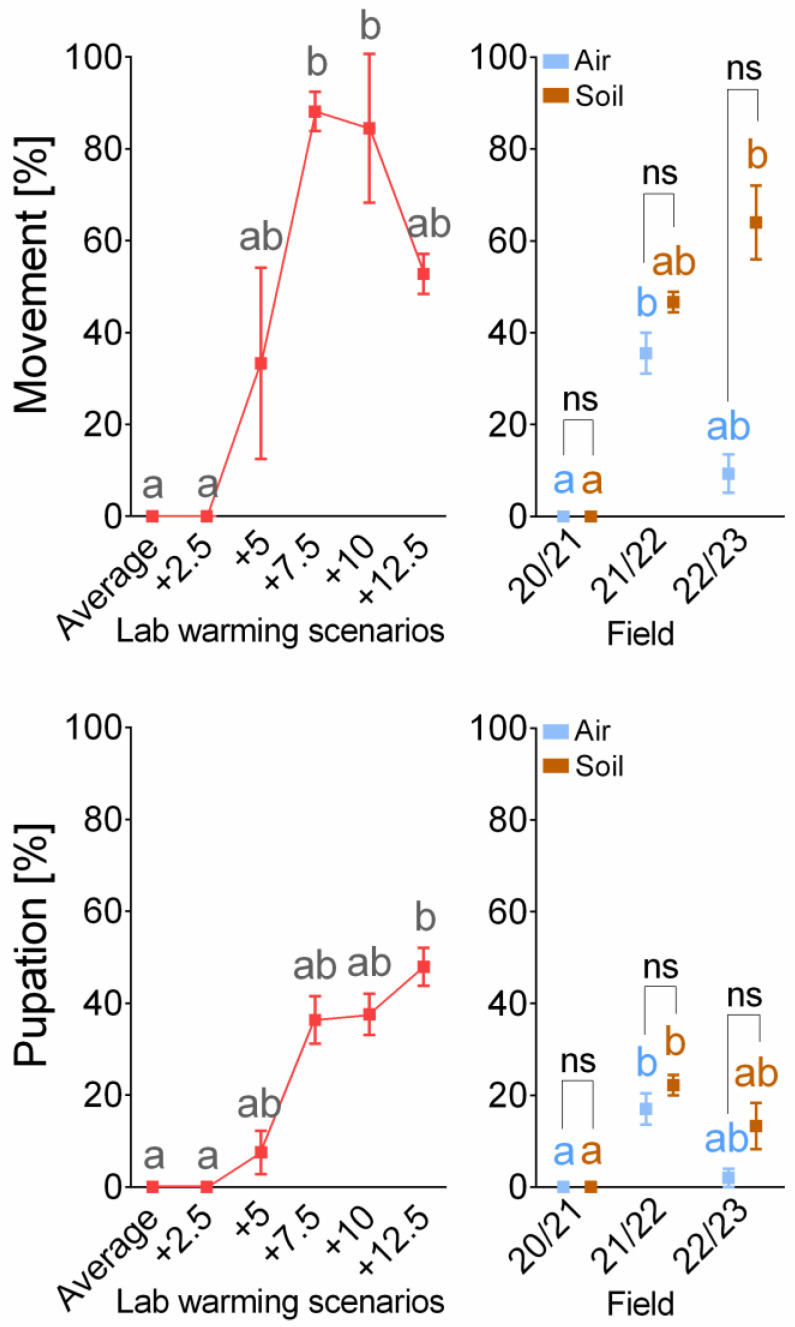
Survival. The figure shows the percentage of larvae able to move and the percentage of larvae able to pupate after exposure to laboratory scenarios and in the field. The “Average” laboratory scenario simulated a long-term average winter in Czechia based on weekly means from 1981 to 2010. The scenarios simulating warming increase the baseline “Average” by 2.5 °C steps, up to “Average + 12.5 °C”. Each point represents mean ± S.D. The effect of treatment on the parameter was tested using the Kruskal–Wallis test, followed by Dunn’s post hoc test. Differences between two groups were tested using Mann–Whitney test. Means marked with different letters are significantly different at *p* = 0.05. The “ns” means no significant difference. Details of statistical analysis are included in Supplementary Table S2.

Survival in the laboratory scenarios (Figure 3, Supplementary Tables S1 and S2) improved with increasing temperature. No larvae survived exposure to scenarios “Average” and “Average + 2.5 °C”. The first signs of survival appeared in the “Average + 5 °C” scenario, where 33.3% of larvae were able to move and 7.5% pupated. The percentages of larvae able to move/pupate in scenarios Average + 7.5 °C, +10 °C, and +12.5 °C were 88.1/36.4, 84.5/37.6, and 52.8/47.9, respectively.

Survival in the field (Figure 3, Supplementary Tables S1 and S2) varied between winters and was higher (although not statistically significantly) in larvae exposed below the soil surface (“Soil”) compared to larvae exposed above the soil surface (“Air”). No larva survived the winter season of 2020/2021, either above or below the soil surface. The percentages of larvae able to move/pupate were 35.6/17.0 above the soil surface and 46.7/22.2 below the soil surface in the season of 2021/2022, and 9.3/2.0 above the soil surface and 64.0/13.3 below the soil surface in the season of 2022/2023.

### 3.2. Temperature Conditions in the Field

Figure 1b shows monthly mean air temperatures at the site of the field experiment during three winter seasons in the period from 2020 to 2023. All three winters were warmer than the long-term average (1981–2010) for Czechia or České Budějovice, which is in line with observations that winter temperatures are indeed rising. Compared to the laboratory treatments, mean air temperatures during the three winters fell between the scenarios “Average +2.5 °C” and “Average + 5 °C”.

Microclimatic temperatures (i.e., temperatures experienced by the larvae exposed in the field; Figure 2, Supplementary Table S4) differed between the three winter seasons of the field experiment. Winter season 2020/2021 was the coldest (close to “Average + 2.5 °C” scenario; no larvae survived), with the lowest temperature of −14.9/−2.0 °C and 46.4/2.5 days below zero in the “Air”/“Soil” treatments, respectively. The following two winters were warmer (closer to “Average + 5 °C” scenario) and also warm enough to allow some larvae to survive. The lowest temperatures and number of days below zero in “Air”/“Soil” treatments were −9.3/−0.8 °C and 32.5/0.1 days in winter 2021/2022, and −6.1/−0.6 °C and 22.1/2.0 days in winter 2022/2023.

## 4. Discussion

This study presents and tests a method for assessing the potential of insect species to colonize colder regions under climate change scenarios. The method is based on exposing insects to warming winter scenarios simulated in the laboratory. The present study tests the method on the example of a Mediterranean pest, *Sesamia nonagrioides,* and asks whether it can survive winters in Central Europe (i.e., in Czechia) if they become warmer. This study also exposes *S. nonagrioides* to real winters under semi-natural conditions in the field.

The results of the laboratory simulations indicate that *S. nonagrioides* can survive winters in Czechia if they warm by 5 °C (possibly even slightly less, as 7.5% of the larvae survived until pupation in the scenario Average + 5 °C) compared to the long-term average from 1981 to 2010. The results of the laboratory simulations are largely supported by the field experiment. Temperatures recorded in the field during three winter seasons in the period from 2020 to 2023 show that all three winters were warmer than the long-term average from 1981 to 2010. The field temperatures during the three winter seasons actually exceeded the estimates by climate models [19]. Field air temperatures during the winter season of 2020/2021 were close to the “Average + 2.5 °C” scenario (Figure 1b), and, as expected, no larvae survived. The following two winter seasons were warmer and brought air temperatures closer to the “Average + 5 °C” scenario, allowing some larvae to survive. The results of the field experiment thus qualitatively support the validity of the simulation experiment. The microclimatic temperatures to which the larvae were actually exposed were even slightly higher than the air temperatures at 2 m above the ground. In seasons 2021/2022 and 2022/2023, some microclimatic temperatures practically reached the temperatures of the laboratory scenario “Average + 5 °C” (Figure 2). The microclimatic temperatures also differed from air temperatures in the range of extremes, which were buffered (to some extent), especially in the soil microclimate (Figure 2).

The statistical test used to analyze the survival data was quite stringent, resulting in many differences between laboratory scenarios and between field treatments being statistically insignificant. On the other hand, if we look more closely at specific cases, we can see that, for example, the difference in survival (i.e., pupation) above the soil surface between the seasons 2020/2021 and 2022/2023 (0% vs. 2%) is statistically insignificant, but from an ecological point of view, it could represent a fundamental difference—extinction vs. survival of the population. Nevertheless, both seasons are likely to produce both results, and the number of replicates would have to be much larger to find exact values. Survival in the field was generally higher in the soil (although not statistically significant), which is in line with the literature, where, for example, Gillyboeuf et al. [16] observed higher survival in larvae overwintering in the underground parts of maize plants. Zero survival in the coldest season of 2020/2021 could have been caused by the extreme temperature in February, when the temperature above the soil surface dropped to almost −15 °C, exceeding the supercooling capacity of the larvae, whose supercooling point (SCP) ranges from −6.5 °C to −9.2 °C [16,17]. However, too low a temperature cannot explain the zero survival of larvae exposed in the soil (the lowest soil temperature in 2020/21 was −2.0 °C), which died for another reason. This could be, for example, the time spent below a critical thermal threshold, at which cold injury can occur.

The results thus indicate that *S. nonagrioides* could overwinter in Central Europe, even under the current state of warming, unless temperature extremes do not fall below the SCP or too cold a winter occurs, exposing the larvae to temperatures below a critical threshold (so far unknown—needs to be investigated) for a prolonged period of time. On the other hand, if the population becomes sufficiently abundant, the probability that some individuals will survive somewhere, even if a temperature extreme occurs, will logically increase. Thus, *S. nonagrioides* could theoretically become established in Central Europe already under current conditions or under slightly warmer conditions in the near future. Similar conclusions were reached by Maiorano et al. [20], who modeled a possible future distribution of *S. nonagrioides* in Europe using a generic phenological model coupled with a model of potential winter mortality. According to their model, *S. nonagrioides* could have the potential to colonize Central Europe by 2030, perhaps even earlier.

This study shows that a simulation experimental approach to the problem of climate change-induced range shifts in insects is feasible and has the potential to provide good estimates. A simplified design of the experiments simulating a single parameter (i.e., temperature; in fact, the photoperiodic regime in Czechia was also simulated) yielded surprisingly accurate estimates for *S. nonagrioides*, as confirmed by the field experiment. Considering that *S. nonagrioides* (and most other insects) are not exposed to air temperatures during overwintering, the reliability of the estimates could be further improved if the simulations were based on microclimatic temperatures, if available. The precision of the estimates also can be improved by appropriately increasing the number of replicates and individuals in the experiments. Although even the simple design appears to work relatively well, more complex simulations can be developed that incorporate changes in other simulable parameters to make the simulations more realistic.

Predicting the effects of climate change on insects and other organisms is challenging. Most current predictions of changes in the distribution of insect species due to climate change typically focus on the growing season and often do not sufficiently consider the responses of organisms to winter conditions [21,22]. Low winter temperatures are often considered to be one of the main barriers to the spread of species from warmer to colder regions [9,10]. However, even if a species can survive winter in a colder region, it may still not be able to colonize that region because some other factor/factors (for instance, inappropriate photoperiodic regime, too high/low precipitation, mismatch between development and food availability, etc.) may not allow that. In order to make more reliable estimates of a species’ potential to colonize a geographical region, conditions (at least abiotic) during both growing and winter seasons should be considered [9,21,23]. Thus, it seems clear that predicting species’ responses to climate change requires consideration of a complex set of parameters over a full year. The method presented in this study could contribute to efforts to predict the effects of climate change on insects, especially during the winter season.

## 5. Conclusions

This study presents a method for predicting the effects of winter climate change on the distribution of insect species. The method is based on laboratory simulations of warming winters and allows us to estimate the potential of a species to survive winters in a given geographical region under a climate change scenario. The applicability of the method is demonstrated using the example of *Sesamia nonagrioides*, whose capacity to survive Central European winters is assessed. By simulating changes in a single parameter (although probably the most important one)—temperature—the method has the potential to provide good estimates of overwinter survival under climate change. The strength of the method is its relative simplicity (i.e., simulation of a single variable—temperature) and the potentially high accuracy of the estimates. The weakness of the method is that it is not a model and is therefore only suitable, on its own, for individual cases. The method may be particularly useful in pest management to estimate changes in overwinter survival and distribution of insect pests due to climate change.

## Data Availability

Data used for the analyses are available in Supplementary Tables S1 and S4.

## Supplementary Materials

The following supporting information can be downloaded at: https://www.mdpi.com/article/10.3390/insects14120957/s1, Table S1: Survival data; Table S2: Statistics; Table S3: Laboratory warming scenarios; Table S4: Field temperatures.
